# Supplementary material for: Diatom lipids open window to past ocean temperatures in the polar regions
Source: Commun Earth Environ. 2026 Jan 9;7(1):154. doi: 10.1038/s43247-025-03177-1 (PMC12893907; doi:10.1038/s43247-025-03177-1)
Supplement: Supplementary file 2 — Supplementary Tables 1 and 2.pdf [file 43247_2025_3177_MOESM2_ESM.pdf]

Supplementary Table 1. EZ_{25} SSTs and related information for cores described in the current study.

BASICC 1

Year (CE)	EZ25 SST (°C)
1996	6.9
1987	7.1
1979	7.5
1970	6.3
1961	7.6
1952	6.3
1943	6.4
1935	7.8
1926	7.0
1917	7.1
1899	7.6
1882	7.7
1864	6.5
1847	8.1
1829	8.2
1785	7.7
1741	6.7

BASICC 43

Year (CE)	EZ25 SST (°C)
1997	3.7
1990	5.1
1982	4.8
1975	4.6
1967	4.6
1959	5.5
1952	5.8
1944	5.3
1937	4.3
1929	5.8
1914	6.0
1898	4.6
1883	4.3
1868	5.2
1853	4.9
1815	4.9
1777	4.8
1739	5.1
1701	5.2

R248 MC010

Year (CE)	EZ25 SST (°C)
2012	10.5
2005	10.3
1996	11.6
1986	9.8
1975	10.1
1963	10.4
1949	11.9
1935	12.8
1919	9.9
1902	10.0
1884	9.8
1864	9.7
1844	9.7
1822	10.5
1799	9.7
1775	10.1
1750	8.8
1723	8.8
1695	8.3
1667	8.6
1637	8.6
1605	7.9
1573	8.5
1540	9.2

MSM5-5-712-1

Year (CE)	EZ25 SST (°C)
1989	10.8
1953	10.0
1917	9.8
1881	9.6
1844	8.8
1808	9.6
1772	9.3
1727	8.0
1700	7.8
1655	7.8
1628	8.9
1582	7.9
1555	8.0
1510	8.5
1483	8.5
1420	8.6
1380	9.1
1313	8.4
1273	8.5
1206	7.9
1166	8.5
1099	9.8
1062	8.6
999	8.0
961	8.1
898	8.4
861	8.3
798	7.4
760	8.7
697	7.6
647	7.9
596	7.9
558	7.7
494	8.6
455	8.5
391	8.1
353	7.3
289	8.1
238	8.8
187	8.7
149	8.3
85	8.1
34	9.4

BC523

Year (CE)	EZ25 SST (°C)	IPSO25/HBI III
2001	2.8	67.7
1994	2.8	60.1
1986	2.8	74.3
1978	3.2	68.5
1969	3.0	60.6
1960	3.1	57.3
1951	3.1	92.9
1941	3.5	190.6
1931	2.9	182.2
1921	2.8	193.7
1910	2.8	204.6
1899	0.5	188.7
1887	1.4	188.8
1875	3.1	204.0

BC660

Year (CE)	EZ25 SST (°C)
2006	4.1
2003	3.7
1998	3.8
1991	3.9
1983	3.8
1972	4.5
1961	4.0
1948	3.8
1933	2.3
1916	3.3
1898	2.4
1878	2.1
1857	2.0
1834	1.9

JM99-1200

Age (cal kyr BP)	EZ25 SST (°C)	IP25 (ng/g sed)
7.26	9.2	0.00
7.47	9.4	0.00
7.77	11.3	0.00
7.98	11.7	0.00
8.39	11.0	0.00
8.69	11.2	0.00
8.90	10.9	0.00
9.20	10.9	0.00
9.41	10.9	0.00
9.61	10.8	0.00
9.82	10.9	0.00
10.02	10.8	0.00
10.22	11.9	0.00
10.63	12.1	0.00
11.12	12.4	0.00
11.47	12.7	0.05
11.55	13.3	0.92
11.59	12.8	0.87
11.61	12.0	1.19
11.62	12.4	0.85
11.63	11.1	0.95
11.64	12.1	1.00
11.64	13.9	1.33
11.65	13.5	1.46
11.65	13.3	0.68
11.66	13.4	1.40
11.66	12.8	1.00
11.67	13.2	1.30
11.67	12.7	1.24
11.68	13.7	1.54
11.68	13.4	1.38
11.69	13.5	1.77
11.70	13.7	2.10
11.70	12.1	2.00
11.70	12.2	1.84
11.71	12.4	1.41
11.72	14.2	1.72
11.72	13.8	1.81
11.72	13.2	2.24
11.73	13.6	1.53
11.74	12.6	0.82
11.78	11.9	1.09
11.78	12.8	1.44
11.80	12.0	2.01
11.81	11.9	2.26
11.82	12.0	2.56
11.83	11.9	2.52
11.84	9.9	2.40
11.85	11.2	3.35
11.86	12.5	5.03
11.87	12.1	4.23
11.88	10.2	7.30
11.89	9.1	11.46
11.90	8.2	19.35
11.91	7.7	18.06
11.92	8.1	18.90
11.93	8.6	17.80
11.94	7.8	19.01
11.95	6.3	17.75
11.96	9.4	21.84
11.97	8.1	18.38
11.98	5.3	17.24
12.03	8.2	16.77
12.14	7.2	19.08
12.17	8.7	19.48
12.26	7.8	19.62
12.46	6.5	19.47
12.59	4.5	16.60
12.61	8.1	19.78
12.62	8.2	21.52
12.65	8.2	23.95
12.66	6.5	25.00
12.67	7.7	21.19
12.70	9.1	21.28
12.73	9.0	19.28
12.74	8.9	22.01
12.75	4.9	23.19
12.76	7.8	21.79
12.76	7.6	21.24
12.77	8.7	17.87
12.78	6.7	21.60
12.78	8.3	19.97
12.79	9.0	19.67
12.80	8.5	19.96
12.80	9.4	19.52
12.81	6.2	17.70
12.81	6.8	16.18
12.83	9.1	16.45
12.83	6.7	16.77
12.84	8.1	15.67
12.84	9.7	17.41
12.85	7.4	15.41
12.86	9.6	15.74
12.86	9.3	15.42
12.87	8.8	13.65
12.88	9.7	11.48
12.90	8.0	9.44
12.91	9.7	11.25
12.93	10.8	6.15
12.94	10.9	10.58
12.98	12.0	0.05
13.04	13.2	0.00
13.09	15.1	0.00
13.14	14.6	0.00
13.19	14.7	0.00
13.24	13.5	0.00
13.32	13.5	0.00
13.41	13.0	0.00
13.49	14.0	0.00
13.58	14.5	0.00
13.66	13.8	0.00
13.74	14.0	0.00
13.82	14.2	0.00
13.87	13.8	0.00

JM09-KA11-GC

Age (cal kyr BP)	EZ25 SST (°C)
0.00	5.4
0.05	5.5
0.16	4.8
0.32	4.8
0.40	5.1
0.45	5.1
0.50	5.2
0.53	5.4
0.57	5.5
0.61	5.8
0.65	6.7
0.69	5.9
0.73	6.2
0.77	6.1
0.81	6.3
0.85	5.7
0.89	6.3
1.18	6.6
1.54	7.9
1.62	6.0
2.09	6.7
2.41	6.5
3.20	6.6
3.36	6.9
3.68	6.6
3.84	6.6
4.15	6.6
4.79	6.4
5.10	6.5
5.34	6.5
5.57	7.3
5.62	6.3
5.67	7.2
5.71	6.7
5.76	6.7
5.81	7.4
5.86	6.6
5.90	7.1
5.95	6.8
6.00	6.9
6.05	7.6
6.09	6.7
6.14	6.8
6.19	7.3
6.27	7.5
6.40	7.6
6.84	7.2
7.09	7.4
7.34	7.5
7.47	7.2
7.61	7.9
7.74	8.1
7.87	7.8
8.00	7.7
8.35	8.1
9.10	8.2
9.90	10.7
9.92	10.5
9.96	12.8
10.00	12.4
10.09	12.6
10.13	12.9
10.15	13.4
10.27	13.0
10.31	12.8
10.35	13.1
10.43	13.4
10.47	12.9
10.51	12.6
10.60	11.6
10.68	10.7
10.72	9.3
10.76	9.1
10.80	9.7
10.84	10.3
10.92	10.5
10.98	11.7
11.41	12.8
11.52	11.1
11.56	11.4
11.60	10.7
11.84	7.6
11.91	9.1
11.95	10.8
12.06	7.5
12.16	7.6
12.27	8.7
12.37	8.7
12.47	7.5
12.58	5.1
12.68	10.3
12.78	9.5
12.89	10.9
12.99	11.4
13.09	10.9
13.12	11.8
13.16	13.1
13.20	12.8
13.24	12.0
13.28	11.7
13.32	10.7
13.36	10.1
13.40	8.3
13.45	13.4
13.62	11.4
13.80	11.3
14.00	11.1
14.20	11.0
14.38	12.5
14.54	12.6
14.74	12.0
14.91	11.2
15.08	7.9
15.43	12.5
15.64	8.3
15.72	7.2

M23258

Age (cal kyr BP)	EZ25 SST (°C)
0.7	9.1
0.9	13.0
1.8	11.8
2.4	11.4
2.7	10.1
3.1	11.0
3.4	10.1
3.7	10.3
4.0	11.5
4.7	12.2
5.2	8.5
5.5	13.8
6.1	11.2
6.8	10.7
7.1	13.5
7.7	10.4
8.0	9.1
8.6	10.7
8.9	11.5
9.3	9.3
9.5	9.1
9.7	8.6
10.0	11.9
10.2	11.2
10.6	12.0
10.8	11.6
11.1	11.9
11.4	12.1
11.8	11.9
12.1	11.5
12.3	9.8
12.5	11.8
12.6	12.6
12.6	11.9
12.7	12.1
12.7	12.1
12.7	12.3
12.8	11.9
12.8	12.8
12.9	11.8
12.9	11.9
13.0	11.6
13.0	11.9
13.1	12.2
13.1	12.1
13.2	11.3
13.3	10.8
13.3	11.0
13.4	11.8
13.5	11.0
13.6	11.9
13.7	12.1
13.8	11.4

NP05-11-70GC

Age (cal kyr BP)	EZ25 SST (°C)
0.0	5.3
0.2	6.5
0.4	6.6
0.5	5.9
0.7	5.9
0.7	6.1
0.9	5.4
1.1	6.8
1.3	5.6
1.4	5.4
1.6	4.8
1.8	6.7
1.9	5.3
2.0	5.3
2.2	5.3
2.3	5.7
2.7	5.8
3.1	5.6
3.5	5.2
3.9	5.7
4.1	5.6
4.3	5.9
4.7	6.7
5.1	6.1
5.5	5.3
5.9	4.4
6.3	5.0
6.5	5.2
6.6	5.3
6.7	5.1
6.9	5.1
7.0	4.7
7.2	4.0
7.3	4.6
7.5	4.5
7.7	5.8
7.8	5.2
8.0	5.5
8.2	5.6
8.3	5.4
8.5	4.6
8.7	5.2
8.8	4.6
8.9	5.2
9.0	5.4
9.1	6.1
9.3	5.2
9.5	6.1
9.6	8.1
9.7	6.5
9.8	
9.9	9.9
10.1	3.6
10.2	7.6
10.2	6.9
10.3	5.9
10.4	5.1
10.5	5.2
10.6	6.0
10.6	5.4
10.7	5.1
10.8	6.6
10.9	4.6
11.1	5.0
11.2	5.0
11.3	4.8
11.4	3.9
11.6	5.4

ODP 1098

Age (cal yr BP)	EZ25 SST (°C)
82	1.8
197	-0.6
299	-1.1
869	-0.5
969	-0.8
1067	-0.2
1096	0.0
1111	-0.8
1154	-0.2
2775	-0.2
2825	-0.6
2875	0.0
3142	-0.7
3183	-0.1
3400	-1.0
3467	0.3
3482	-0.4
3492	-0.7
3611	0.4
3830	-0.4
3950	-0.9
4066	-0.3
4343	-0.9
4655	-0.4
4692	1.2
4711	-0.2
4732	-0.1
4854	-0.5
5016	-0.4
5177	-0.6
5213	0.4
5252	0.1
5372	-0.1
5413	-0.1
5457	0.0
5493	-0.2
5589	0.1
5712	-0.4
5712	-1.1
5738	0.1
5835	0.5
5873	-0.5
5911	0.3
5931	-1.0
5996	0.3
6025	0.3
6069	-0.1
6075	-0.8
6504	-0.2
6804	-0.7
6868	1.7
6905	1.1
7143	-0.9
7245	-0.9
7567	-0.5
7969	0.2
7976	0.8
7980	-0.1
8015	0.4
8338	-0.3
8364	1.8
8412	0.9
8457	0.5
8502	0.2
8654	-0.7
8876	-0.2
8900	-0.1
8949	0.3
9571	-0.6
9896	0.1
9918	0.0
9942	0.2
10018	-0.3
10525	-0.1
10502	0.0
10616	-0.2
10690	0.5
10773	0.5
10878	6.0
10902	0.2
10925	1.2
10951	4.5
10977	0.8
11001	3.2
11025	2.5
11027	0.0
11051	0.1
11088	0.0
11137	-0.2
11159	-0.1
11472	-0.1
11634	0.1
11678	-0.7

ODP 910A

Age (years*1000)	Depth (mbsf)	EZ25 SST (°C)	IP25 (ng/g)	Dinosterol (µg/g)	Brassicasterol (ng/g)	Spring sea ice concentration (%)
8.57	0.15	4.24	0.21	1.59	88.87	44.51
14.29	0.25	4.26	1.52	11.97	831.1	25.53
20.00	0.35	4.73	0.26	1.46	80.06	58.54
23.75	0.45		0.00	0.10	4.96	
27.50	0.55		0.00	0.19	8.38	
31.25	0.65		0.00	0.12	6.33	
35.00	0.75	3.18	0.34	2.55	152.29	37.10
38.75	0.85	3.36	1.47	4.86	430.17	31.76
42.50	0.95	3.39	0.96	4.15	326.35	32.80
46.25	1.05	3.50	1.17	4.27	361.45	31.01
50.00	1.15	3.71	1.03	2.98	313.48	33.64
53.75	1.25	3.17	0.98	4.44	376.94	32.80
57.50	1.35	3.50	1.35	6.23	388.22	33.76
65.17	1.55	3.67	0.96	4.04	389.1	25.08
69.13	1.65	3.73	1.12	3.96	346.4	32.46
77.04	1.85	3.56	1.10	3.55	383.96	30.42
85.36	2.15	2.83	0.93	3.94	405.49	28.40
90.80	2.40	3.27	1.30	3.85	397.99	30.02
96.24	2.65	3.48	1.16	6.28	396.79	29.74
103.87	3.00	2.82	1.17	5.43	571.96	27.79
107.13	3.15	3.25	1.60	4.45	520.99	27.35
113.67	3.45	3.18	1.12	4.39	437.96	30.98
118.02	3.65	3.48	1.13	4.90	384.07	31.74
122.38	3.85	3.51	1.03	2.68	285.09	29.32
126.73	4.05	3.21	1.52	8.58	569.75	30.73
131.67	4.25	3.60	1.22	4.24	394.85	29.81
141.61	4.55	3.44	1.29	2.93	304.84	31.18
150.93	4.84	3.13	1.13	5.47	594.47	29.29
160.89	5.15	3.17	1.27	3.74	413.16	29.49
166.04	5.31	3.10	1.31	6.95	465.83	29.13
173.75	5.55	3.62	1.12	4.30	403.25	27.01
179.54	5.73	3.50	1.19	5.47	409.09	30.68
183.39	5.85	3.49	1.56	3.83	431.46	28.22
192.97	6.05	3.37	1.43	5.18	506.61	28.25
198.28	6.15	2.89	1.64	5.78	671.86	26.81
208.91	6.35	3.36	1.19	4.75	393.47	29.33
219.53	6.55	3.71	1.13	3.50	368.69	30.44
224.84	6.65	3.35	1.08	5.01	435.81	30.98
232.81	6.80	3.09	1.23	6.96	487.64	28.06
240.78	6.95	3.16	1.14	4.75	436.77	30.69
246.09	7.05	3.06	1.40	5.90	549.21	29.05
251.41	7.15	3.08	0.89	4.45	432.67	28.73
262.03	7.35	3.18	1.47	5.87	542.02	28.78
267.34	7.45	3.41	1.28	4.74	496.87	28.01
282.71	7.65	3.38	1.24	4.86	440.55	30.34
291.18	7.75	3.47	1.15	4.76	492.76	28.19
299.65	7.85	3.50	1.54	5.13	463.48	30.07
308.12	7.95	3.10	1.46	5.90	561.69	29.01
316.59	8.05	3.33	1.25	3.99	410.04	28.47
325.06	8.15	3.32	1.60	5.18	503.55	30.35
333.53	8.25	3.51	0.82	6.71	390.5	31.75
348.56	8.45	3.44	0.93	4.40	428	28.62
355.12	8.55	3.34	1.20	5.34	492.89	28.44
364.96	8.70	3.32	1.34	3.87	428.87	27.83
374.80	8.85	3.33	1.34	5.61	525.91	28.27
381.36	8.95	3.29	1.19	4.85	522.56	27.13
397.76	9.20	3.14	1.42	5.33	539.97	26.34
404.32	9.30	3.19	1.50	4.31	467.84	28.60
417.44	9.50	2.75	1.68	7.07	468.07	29.88
425.25	9.70	2.96	1.27	4.99	513.82	30.41
430.25	10.10	3.40	0.82	2.46	217.58	29.28
432.75	10.30	3.28	1.03	4.26	427.05	28.45
443.98	10.60	3.16	1.02	3.81	418.48	27.48
450.62	10.85	3.42	1.24	7.24	424.12	31.75
457.25	11.10	3.28	1.35	4.96	510.8	27.87
462.56	11.30	3.39	1.31	5.33	484.07	28.72
465.22	11.40	3.35	1.27	3.72	406.59	29.71
473.18	11.70	3.21	1.29	4.31	482.49	28.96
478.49	11.90	3.01	1.77	5.29	622.01	28.26
483.80	12.10	3.20	1.00	6.63	404.63	28.74
489.11	12.30	3.12	1.26	5.63	511.41	27.38
494.42	12.50	3.53	1.41	3.95	421.67	28.47
499.73	12.70	3.47	1.23	5.08	448.38	29.02
505.04	12.90	3.20	1.24	4.73	464.17	27.77
513.00	13.20	3.22	1.36	4.34	480.88	27.41
515.66	13.30	3.30	1.14	7.07	433.85	30.68
523.64	13.60	3.21	1.27	5.55	510.31	28.03
528.96	13.80	3.62	1.30	4.20	470.31	29.59
534.28	14.00	3.46	1.22	5.24	467.81	27.61
536.94	14.10	3.30	1.31	4.15	475.44	29.47
540.08	14.34	3.21	1.27	4.31	476.02	27.13
548.00	15.10	3.51	1.29	7.05	435.49	30.68
555.50	15.30	3.25	1.40	5.05	495.39	26.06
559.25	15.40	3.23	1.26	6.05	534.11	27.01
563.00	15.50	3.07	1.59	5.34	509.72	27.81
569.11	15.60	3.51	1.46	3.37	407.84	30.35
581.32	15.80	3.52	1.06	4.48	424.46	30.09
587.42	15.90	3.49	1.62	5.26	514.88	31.26
593.53	16.00	3.28	1.33	4.89	441.97	31.18
599.63	16.10	3.04	1.11	5.22	485.7	30.65
605.74	16.20	2.89	1.73	5.98	581.18	31.50
611.84	16.30	2.95	1.44	5.64	684.87	28.00
617.95	16.40	2.91	1.93	7.13	734.19	27.30
627.88	16.50	2.73	1.91	7.12	789.59	29.47
655.38	16.70	2.82	0.13	1.14	59.6	27.83
669.13	16.80	3.12	1.07	4.70	361.83	31.42
677.29	16.90	3.33	0.82	3.33	392.49	29.67
679.87	17.00	3.20	1.13	5.31	489.64	28.15
682.44	17.10	3.25	1.50	5.21	485.68	29.28
685.02	17.20	3.10	1.66	5.02	473.34	29.74
687.60	17.30	3.23	1.26	7.77	473.16	29.51
690.17	17.40	3.42	1.56	4.98	492.32	29.83
692.75	17.50	2.86	1.40	4.89	491.2	27.44
695.33	17.60	3.10	1.72	6.12	643.04	28.48
697.90	17.70	3.37	1.33	5.85	562.45	28.51
700.74	17.81	3.23	1.62	4.39	498	27.80
703.06	17.90	3.36	1.60	5.46	533.67	28.32
705.63	18.00	3.30	1.40	5.47	558.82	27.80
708.21	18.10	3.26	1.19	4.08	498.65	27.08
713.36	18.30	3.09	1.41	5.51	519.85	28.23
715.94	18.40	2.91	1.22	4.32	469.26	28.81
719.30	18.50	3.11	1.69	5.73	571.16	28.21
725.79	18.60	2.73	1.24	7.25	468.11	29.48
732.29	18.70	3.22	1.38	4.64	458.62	28.77
738.78	18.80	2.91	1.29	4.83	495.84	25.05
745.28	18.90	3.00	1.59	5.40	536.56	27.06
751.77	19.00	2.98	1.49	5.41	516.93	28.78
758.92	19.11	3.11	1.65	4.46	520.11	28.80
768.66	19.26	3.01	1.29	4.93	569.71	26.58
778.40	19.41	2.82	1.40	5.49	515.86	31.08

Supplementary Table 2. ^{210}Pb data and age models for BC 660 and BC 523.

BC 660

Depth (cm)	Age (cal kyr)	Age (yr)					Predicted CRS dates from		CRS Model		
				Core	Mid depth (cm)	210Pb	Predicted Cum Pb210	Dates (Jeter 2000)	Pb210 Age	poly2 reg line	poly3 reg line
0.5		2012				dpm/g (calc)					
1.5		2008		BC660	0.5	26.43	2007.6	2007.5	2008.3	2006.3	2009.7
2.5		2002		BC660	1.5	29.13	2002.6	1998.4	2003.0	2002.8	2002.6
3.5		1997		BC660	2.5	25.80	1996.7	1989.3	1997.1	1997.7	1995.6
4.5		1990		BC660	3.5	26.15	1990.1	1980.2	1990.0	1990.9	1988.5
5.5		1982		BC660	4.5	22.65	1982.5	1971.1	1981.5	1982.5	1980.7
6.5		1973		BC660	5.5	21.91	1973.8	1962.0	1971.3	1972.5	1971.8
7.5		1961		BC660	6.5	19.23	1963.9	1952.9	1958.6	1960.8	1961.6
8.5		1949		BC660	7.5	13.59	1952.8	1943.8	1947.0	1947.6	1949.5
9.5		1936		BC660	8.5	9.94	1940.1	1934.7	1933.9	1932.7	1935.2
10.5		1923		BC660	9.5	6.63	1925.6	1925.6	1921.7	1916.1	1918.2
11.5		1906		BC660	10.5	5.68	1908.5	1916.5	1903.6	1898.0	1898.3
12.5		1874		BC660	11.5	5.01	1887.3	1907.5	1870.7	1878.2	1874.9
13.5		1839		BC660	12.5	2.94		1898.4		1856.8	1847.7
					13.5	0.00					

BC 523

Depth (cm)	Age (cal yr BP)	Age (yr AD)					CRS Model		
				Core	Mid depth (cm)	210Pb	Pb210 Ages	poly2 reg line	poly3 line
						dpm/g (calc)			
1.5	0	2003		BC523	0.5	25.82	2006.0	2007.9	2007.8
2.5	4	1999		BC523	1.5	21.90	2000.9	2001.0	2001.0
3.5	10	1993		BC523	2.5	26.10	1995.2	1993.6	1993.8
4.5	18	1985		BC523	4.5	27.24	1981.3	1977.8	1978.0
5.5	30	1973		BC523	6.5	16.23	1959.6	1960.4	1960.6
6.5	43	1960		BC523	8.5	5.06	1937.6	1941.5	1941.6
7.5	57	1946		BC523	10.5	2.94	1920.7	1920.9	1921.0
8.5	70	1933		BC523	12.5	2.40	1900.7	1898.8	1898.8
9.5	78	1925		BC523	14.5	0.94	1875.1	1875.1	1875.1
10.5	86	1917		BC523	16.5	0.47	1849.1	1849.8	1849.8
11.5	96	1907		BC523	18.5	0.20	1824.1	1822.9	1823.1
12.5	110	1893		BC523	20.5	0.12	1793.8	1794.5	1794.9
13.5	129	1874		BC523	22.5	0.08			
14.5	153	1850							
